# Our Defences

**Published:** 1857-02

**Authors:** 


### HALL'S JOURNAL OF HEALTH.

OUR LEGITIMATE SCOPE IS ALMOST BOUNDLESS: FOR WHATEVER BEGETS PLEASURABLE AND HARMLESS FEELINGS, PROMOTES HEALTH; AND WHATEVER INDUCES DISAGREEABLE SENSATIONS, ENGENDERS DISEASE.

VOL. IV.] FEBRUARY, 1857. [NO. II.

We aim to show how disease may be avoided, and that it is best, when sickness comes, to take no medicine without consulting an educated physician.

### OUR DEFENCES.

We have occasionally seen exceptions taken to some of our teachings, and no doubt others have occurred which have not met our eye. We wish to inform our readers, once for all, we write for utility. Nothing is radically and permanently useful which is not true; hence it is our effort always to be literally correct in all our statements, and those statements are made either as the result of our own observation or the *corroborated* observations of scientific men recorded in standard publications. Now with this inflexible rule and object before us, it is not a very difficult matter to issue a publication monthly which merits public reliance. Of all the criticisms of our *Journal*, the one of more frequent occurrence north, south, east and west, is "*its frank, strong common sense!*" Whence comes this descriptive phrase? It arises from the fact that our teachings correspond with the observation, experience and rationality of the reader. We do not advocate an idea simply because a scientific man eliminates it, for it is a commonly observed fact that scientific men are practically men of the least every-day sense. A man may be a scientific fool. Nor do we write from the experience of a day, but from the uniform experiences of an eventful life, and from the opportunities of wide research and observation. It was Count Rumford who deduced scientifically that white clothing was the warmest in winter, and increased his reputation for oddity by dressing in white during the coldest of New England winters, but he made no impression of his views on the minds of the every-day matter of fact people about him.

"I knows what I knows, and I knows what I don't know," said a daft youth to the village miller, who twitted him as to his scanty knowledge. "Indeed," answered the miller, "how is that?"

"Why, I know your pigs are fat, but I don't know whose corn they eat."

Many persons write for the newspapers merely from the store of knowledge they possess, irrespective of what they do not know, and hence a host of crude contributions. The knowledge of many is of a kin in quality, to that of the man who had never seen any land beyond the little island which he had not left from the hour of his birth; he knew that was the only land in the world—because he had seen no other. A fact may come to our knowledge, and we may draw a legitimate conclusion, but additional knowledge may show that it was only part of a fact, and what may be a wise deduction from a part may be a very foolish one when drawn from a whole.

We trust, therefore, that our readers will not conclude us in error from the mere fact that our statements are controverted. The daily and weekly papers very often presume opinions on medical subjects, from the modicum of intelligence which they possess in that direction, whereas if they had known a little more, they would have been ashamed of their immaturity.

### MORNING WALKS MISCHIEVOUS.

Under the head of *Popular Fallacies* we stated, two years ago, that while early rising was commendable, it was better to remain in bed to a late breakfast and then go to work, than to go out of doors for any purpose before breakfast, especially in the summer time, in the west and south-west. It is injurious to the health of anybody in any climate, in any season of the year, to go to any out-door occupation. Some persons may do it with comparative impunity by reason of vigorous health and an active life, but scientific deductions, in common with the ordinary observations of men, are conclusive of the fact, that in all seasons and climates, those who are engaged in out-door occupations, or have to leave their houses early in the morning, ought to eat a regular breakfast before going out; and if that is not practicable, to eat at least a crust of bread or an apple in order to wake up the stomach to an activity which can repel the hurtful influences of cold in winter, and of that *malaria* in summer, which is more or less malignly present about sunrise, wherever a tree flourishes or a blade of grass springs. These are facts known to every educated physician in the world. And yet a contemporary exclaims violently, on the ground of his having been taught from infancy that early rising was healthful,—"Is there no truth?" And to make our assertion appear self-evidently ridiculous he cries out, "Are not birds healthy, and they sing joyously at sunrise?" But to say nothing of the fact that we were speaking as to men and not about brute beasts, a moment's reflection might have suggested, that while it was true that birds had good health and were on the wing early, yet it was not reasonable to suppose that a bird, no more than a man, is inclined to song or mirth on an empty stomach. Besides, as far as our observation has extended among the multitudinous chickens which our mother would always have about her in town and country, not one of them, from the most venerable rooster down to the youngest chicken, ever failed to fly from the tree to the trough. The first instinct of a chick as well as a child is to get a breakfast.

It is not healthy anywhere to get up early and go to work unless something is first eaten. Thousands die every year, and other thousands do more than that, shake with the ague for years in new countries, from failing to eat breakfast before they go out to work. It is useful to observe, that national habits are often the result of their uniformly observed propriety. So what is not healthful in one nation may be in another. Thus it is that the instincts of different nations establish various kinds of food as favorites. Bread is the favorite of the French; maccaroni of the Italian; rice of the Chinese; roast beef of the English; sauerkraut of the German; molasses and pumpkin pie of the New Englander; corn bread of the Kentuckian. The Irishman feasts on his potatoe, while Sawney revels in oatmeal porridge. In the numerous milk-and-water books of travel which annually afflict the reading public, the writers give full frequent evidence of their incapacity, by slighting remarks made of the domestic habits and customs of the people, making their own the standard of perfection, and not taking into judicious consideration the different surroundings of the people among whom they travel.

*The Best Tooth Wash*, because the safest, most familiar, and most universally accessible, and most invariably applicable and efficient, where specific dental science is not sought, is a piece of common white soap with a brush of moderate stiffness. The correspondent of a medical cotemporary inquires as to the truth of the statement, to which the editor replies simply, "*It is nonsense!*" What are the ascertained facts of the case? "Tartar on the teeth," is a familiar expression. Microscopical examination shows that millions of living things are there,—that there are mainly two kinds, and that the larger class are instantaneously killed by soap-suds, when strong acids have no effect whatever. Here is a simple fact, on which eminent dentists have based the practical advice to use common white soap as a corrector and preventive of tartar on the teeth to a considerable extent, so that we almost look contemptuously on the flippancy of an editor who would write "nonsense" to such a statement, when most probably he never made a microscopical examination of this "tartar," and never performed an experiment on the living things which seem to form it from a product furnished by the animal economy, very much perhaps as coral is formed by myriads of invisible creatures from material found in the waters of the sea.

*Corns Cured.*

We stated in another number, that the simplest, safest, most available, and consequently the best cure for corns on the toes, consisted in three operations,—First: Soak the feet in hot water for fifteen minutes, night and morning, for a week.

Second: After the soaking, rub a little sweet oil on the corn, or any other mild form of grease, with the finger, for about five minutes.

Third: Cut a hole in one, two, or three thicknesses of soft buckskin, and bind it on the toe, so that the hole in the buckskin shall receive the corn.

The object of the water and oil is to soften the corn and parts adjoining; the object of the buckskin is to protect the corn from pressure.

In a very short time the corn will become painless, and will subsequently fall out of itself, as it is a growth, and is pushed upwards and outwards by the more natural growth beneath. It is thrown out of the body by the action of the parts, as a splinter or a crushed bone, as being no longer a part of the body. If anything else is done to the corn, it should be simply picked out with the finger nail, as cutting it makes it take deeper root, and dangerous bleedings sometimes occur when the knife is used. Our object was to recommend something which we knew to be safe, practicable, and efficient. We stated further, that corns, like consumption, were never cured; and that the original causes of them, to wit, pressure with a tight shoe, or friction with a loose one, would cause them to return, and even more readily than at first, because the structure of the skin about a corn is malformed for life, and would no more become entirely natural than a finger would grow again, if cut off. So, when a person has had consumption and gets well, he cannot be said to be perfectly cured, because he will always remain with a deficiency of lungs; the portion destroyed never can be replaced, yet, by making the remainder of the lungs work more fully, he may have even better health than he ever had before. Thus, it is literally and strictly true, that corns and consumption are never cured—because their very existence depends on the destruction of the organization and substance of a part of the body, never to be replaced.

There are corn doctors and corn salves; some of which latter are of very powerful constituents. They do remove the corns, but for no longer time than the plan we have advised, while ours has the preference, as costing nothing, and being wholly without danger. Remedies are valuable for the multitude in proportion as they are safe, cheap, and attainable.

If corns are pared down, from time to time, they widen their base, become more deeply rooted, and in old age blacken, cause gangrene and ultimate death! Therefore, we advise the prompt treatment of corns as above. It may be many months before they return; but when they do, the same treatment will always be effectual, and always safe.

